# Overrepresentation of human epidermal growth factor receptor 2 positive- and Luminal B breast cancer metastases in the eyes and orbit

**DOI:** 10.1038/s41433-022-02363-1

**Published:** 2022-12-14

**Authors:** Gustav Stålhammar, Hans E. Grossniklaus

**Affiliations:** 1 Department of Clinical Neuroscience, Division of Eye and Vision, St. Erik Eye Hospital, Karolinska Institutet, Stockholm, Sweden; 2 St. Erik Eye Hospital, Stockholm, Sweden; 3 Departments of Ophthalmology and Pathology, Emory University School of Medicine, Atlanta, GA USA

**Keywords:** Predictive markers, Metastasis

## Abstract

**Background:**

Breast cancer is the most common cancer to spread to the choroid and orbit. Depending on a set of prognostic and predictive biomarkers, breast cancer can be divided into at least four distinct subtypes with separate treatment and clinical course.

**Subjects:**

Thirty-two patients with metastases to the eye and periocular area diagnosed between 2005 and 2020, of which 11 also had primary tumour tissue available. Expression levels of oestrogen- (ER) and progesterone receptors (PR), Human epidermal growth factor receptor 2 (HER2) and the proliferation marker Ki67 were analysed.

**Results:**

Twenty-five of 32 patients (78%) had a history of primary breast cancer, whereas the remaining 7 (22%) presented with metastatic disease. Of available metastases, 83% were positive for ER, 37% for PR, 54% for HER2, and 50% for Ki67. Metastases had significantly lower proportions of PR-positive cells than primary tumours, and the distribution of the Luminal A, Luminal B, HER2 enriched and triple-negative subtypes differed between primary tumours and metastases (*P* = 0.012): Six of 9 patients with a full set of biomarkers on both primary tumours and metastases switched subtype (67%), and 23 of 32 metastases (77%) were of the Luminal B subtype.

**Conclusions:**

Nearly 4 in 5 breast cancer metastases in the eyes and orbit are of the Luminal B subtype, and a majority are HER2 positive. The breast cancer subtype frequently switches between primary tumours and metastases. Future studies should evaluate these results in larger cohorts.

## Introduction

One in eight U.S. women will be diagnosed with breast cancer during her lifetime. For women worldwide, it is the most common cause of cancer death after lung cancer [1].

Breast cancer is classified histologically according to morphological characteristics [2]. For example, invasive carcinoma of no special type (NST), formerly known as invasive ductal carcinoma, typically grow in clusters or gland-like structures whereas invasive lobular carcinomas often grow in single files of cells. Among women with metastatic breast cancer, lobular histology is associated with worse overall survival [3]. However, the morphological patterns provide no detailed prognostic information or guidance on treatment.

For the last two decades, breast cancer has therefore been classified according to molecular characteristics, with proven prognostic and predictive value [4, 5]. The hormone receptor-positive subtypes Luminal A and Luminal B constitute 45 to 65 and 20 to 26% of all primary breast cancers, respectively [6–8]. Luminal A breast cancer implies a relatively good prognosis and patients can usually be treated with surgery and hormonal therapy alone, whereas Luminal B is more aggressive and may require adjuvant cytotoxic chemotherapy, depending on anatomic extent [9]. Human epidermal growth factor receptor 2 (HER2) amplified carcinomas, constituting 10 to 30% of primary tumours and about 30% of metastases, are aggressive but can be treated with monoclonal antibodies (Trastuzumab) [4, 10, 11]. Triple-negative carcinomas do not express hormone receptors and are not HER2 amplified, which means that treatment options are relatively limited and that they are associated with a poor prognosis [7, 9].

Previous studies have found that some of these variants have a tendency to metastasize to certain parts of the body. For example: Triple negative metastases are overrepresented in the brain [12]. Additionally, it has been shown that a switch in subtype can occur between the primary tumour and the metastasis, and that the sampling method is important for the result, with discrepancy in biomarker status between fine needle aspiration cytology and core needle biopsies [13, 14].

Breast cancer is the most common cancer to spread to the choroid of the eye and to the orbit, which entails a poor patient prognosis and a median overall survival of approximately 1 to 3 years [15–17]. Orbital metastases from breast cancer tend to infiltrate extraocular muscles and fat, impairing eye motility [17]. Alternatively, scirrhous infiltration can occur, which typically leads to enophthalmos [17].

To the best of our knowledge, it has not been investigated if one or several of the breast cancer subtypes are overrepresented or if there is a tendency for the subtype to switch in this anatomical area.

## Methods

All patients diagnosed with breast cancer metastasis in the eyes, orbits and eyelids at the L.F. Montgomery Laboratory and the Ocular Oncology and Pathology service, Emory Eye Center, Atlanta, GA, USA and at the St. Erik Ophthalmic Pathology Laboratory, St. Erik Eye Hospital, Stockholm, Sweden between 2005 and 2020 were considered for the study. A total of 107 patients were identified, of which 40 had glass slides available in our archives. Nineteen of the 40 available cases (48%) and 49 of the 67 unavailable cases (73%) had been diagnosed before 2015. The plausible major reason for the missing cases was therefore that they were older and had been discarded from the archives, sent back to home clinics or similar. Of the 40 available cases, 8 had not been stained with the full panel of biomarkers required for surrogate subclassification of breast cancer: oestrogen receptors (ER), progesterone receptors (PR), HER2, and the proliferation marker Kiel 67 (Ki67). Thirty-two patients remained for analysis. From these patients, we had access to data from pathology reports and medical journals on age, sex, metastatic locale, method for tumour sampling, and date of primary breast cancer diagnosis. For 11 patients, we also had access to data on the primary tumour including results of immunohistochemical stains and/or fluorescence in situ hybridization (FISH) for centromere 17 and the *HER2* gene. This study adhered to the tenets of the Declaration of Helsinki and was approved by The Regional Ethical Review Board in Stockholm (reference 2018/2077-32) and the Institutional Review Board of Emory University (reference IRB00107119). Both boards waived informed consent as this was a retrospective study based on already collected data, that did not affect the diagnostic work-up, treatment or follow-up of patients, and did not require new tissue sampling or processing.

### Immunohistochemical staining and FISH

All American metastases had been paraffin-embedded, sectioned and stained at the L.F. Montgomery Laboratory and all Swedish metastases at the St. Erik Ophthalmic Pathology Laboratory. Paraffin blocks were cut into 4 µm sections, pre-treated in ethylenediaminetetraacetic acid (EDTA) buffer at pH 9.0 for 20 min and incubated with mouse monoclonal antibodies against CkAE1/3 (ThermoFisher Scientific, Waltham, MA, USA) and rabbit monoclonal primary antibodies (Ventana, Oro Valley, AZ, USA) for ER (clone SP1), PR (clone 1E2), Ki67 (clone 30-9), and HER2 (clone 4B5) according to the manufacturers’ instructions, and finally counterstained with haematoxylin and rinsed with deionized water. The deparaffinization, pre-treatment, primary staining, secondary staining, and counter-staining steps were run in a Bond III automated IHC/ ISH stainer (Leica, Wetzlar, Germany) at both institutions. The dilutions had been gradually titrated until optimal staining was achieved, according to manual control.

Dual-probe FISH for centromere 17 and the *HER2* gene had been performed with kit assays (PathVysion, Abbott SAS, Rungis, France) according to the manufacturer’s instructions. The DNA probes and tissue sections were denatured for 5 min at 85 °C with a HYBrite instrument (Abbott) and counterstained and mounted with a solution of 4,6-diamidino-2-phenylindole (DAPI). Both authors viewed and scored the slides in consensus.

### Surrogate subclassification

The assessments of ER, PR, HER2, and Ki67 were combined and compared for classification into surrogate immunohistochemical subtypes for each tumour. Based on recommendations from international expert consensus, we used a threshold of ≥1% of tumour cells for ER positivity, and ≥20% of tumour cells for PR positivity [18–20]. Tumours were classified as positive for HER2 IHC if membranous staining was observed in a homogeneous and contiguous population of at least 10% of the tumour cells; or for HER2 FISH if the single probe average *HER2* copy number was ≥6.0/cell [21, 22]. For Ki67, we applied a threshold of >25%, based on previous studies in which a cutoff of 19 to 35% has been shown to be prognostically relevant in our setting (Table 1) [6, 23, 24].

**Table 1:** Intrinsic breast cancer subtypes and surrogate definitions by immunohistochemical profile.

Intrinsic subtype	Surrogate IHC classification
Luminal A	ER ≥ 1% and PR ≥ 20% and HER2 ‘negative’ and Ki67 ≤ 25%
Luminal B	1. ER ≥ 1% and/or PR ≥ 20% and HER2 ‘negative’ and Ki67 > 25%, or
	2. ER ≥ 1% and PR < 20% and HER2 ‘negative’ and any Ki67, or
	3. ER ≥ 1% and/or PR ≥ 1% and HER2 ‘positive’ and any Ki67
HER2-enriched	ER < 1% and PR < 1% and HER2 ‘positive’ and any Ki67
Triple-negative	ER < 1% and PR < 1% and HER2 ‘negative’ and any Ki67

*ER* Oestrogen receptor, *PR* Progesterone receptor, *HER2* Human epidermal growth factor receptor 2. *IHC* Immunohistochemistry. ‘%’ refers to the proportion of tumour cells unambiguously stained with the respective biomarker. ‘Positive’ and ‘negative’, as defined by the American Society of Clinical Oncology and College of American Pathologists recommendations for human epidermal growth factor receptor 2-testing in breast cancer.

### Statistical methods

*P*-values below 0.05 were considered statistically significant, all *P*-values being two-sided. For tests of continuous variables that did not deviate significantly from normal distribution (D’Agostino-Pearson omnibus normality test *P* > 0.05) Student’s *t*-tests were used. For non-parametrical data, Mann–Whitney *U* tests were used. For comparisons of categorical variables, we used contingency tables and Pearson chi-square (χ^2^) tests (if all fields had a sample of >5) or Fisher’s exact tests (if any field had a sample of <5). The Sankey diagram in Fig. 2 was made with SankeyMATIC (sankeymatic.com). All statistical analyses were performed using IBM SPSS statistics version 27 (Armonk, NY, USA) and GraphPad Prism version 9.3.0 (San Diego, CA, USA).

## Results

### Descriptive statistics

Of the 32 included patients, all were women. Twenty-five patients (78%) had a history of primary breast cancer. For the remaining 7 (22%), the ocular metastasis was the first presentation, and the breast cancer was only diagnosed after biopsy and subsequent systemic evaluations. Nineteen patients (59%) had metastases at other sites at the time of presentation, whereas 13 patients (41%) had no other known metastases. Most ocular metastases were located in the orbit, with fewer metastases in the eyelids and choroid. The median time elapsed between the diagnosis of primary breast cancer and metastasis was 3 years (Table 2). All examined continuous variables (proportion of ER, PR and Ki67 positive cells) deviated from normal distribution (D’Agostino-Pearson omnibus normality test *P* > 0.05).

**Table 2:** Demographics and clinical features of study patients.

*n*	32
**Age at metastasis, mean (SD)**	63 (12)
**Sex,** ***n*** **(%)**	
Female	32 (100)
Male	0 (0)
**History of primary breast cancer,** ***n*** **(%)**	
Yes	25 (78)
No	7 (22)
**Metastasis location,** ***n*** **(%)**	
Orbit	19 (59)
Choroid	6 (19)
Eyelid	6 (19)
Extraocular muscle	1 (3)
**Sampling method,** ***n*** **(%)**	
Incisional biopsy	25 (78)
Core needle biopsy	3 (9)
Enucleation	2 (6)
Excisional biopsy	1 (3)
Transvitreal biopsy	1 (3)
**Other metastatic sites,** ***n*** **(%)**	
Bone only	5 (16)
Lymph nodes only	3 (9)
Bone and liver	2 (6)
Subcutaneous only	2 (6)
Peritoneum only	2 (6)
Lymph nodes and mediastinum	2 (6)
Bladder only	1 (3)
Lung, brainstem and brain	1 (3)
Endometrium	1 (3)
No other metastases	13 (41)
**Median time primary breast cancer diagnosis to metastasis, years (IQR)**	3 (3)

*SD* standard deviation. *IQR* interquartile range.

### Biomarker status in primary tumours and metastases

**Fig. 1:**
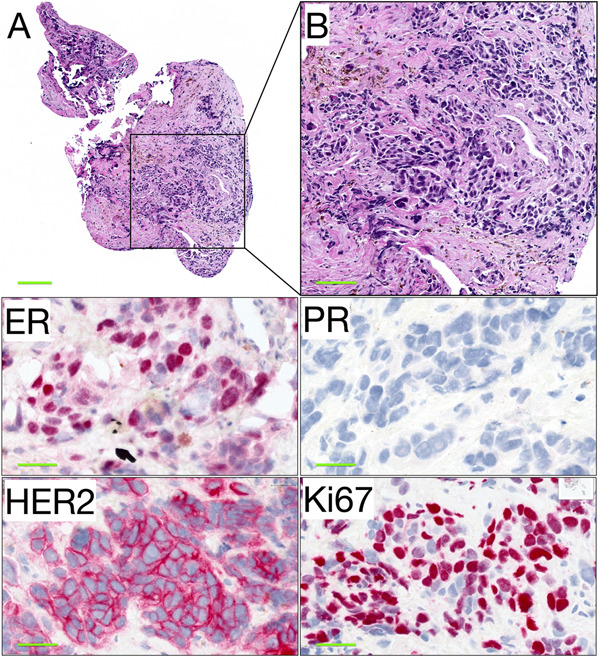
Transvitreal incisional biopsy from a choroidal metastasis of breast cancer. **A** Irregular clusters of enlarged epithelioid cells grow in choroidal tissue with severe fibrotic changes, which may be a reaction to tumour infiltration and inflammation. **B** In larger magnification, the infiltrating tumour cells are seen with pleomorphism, hyperchromasia, and a tendency to form rounded gland-like structures. Most of the tumour cell nuclei were positive for ER (red, chromogen), but negative for PR (blue, haematoxylin). There was complete and intense membranous HER2 positivity in all tumour cells. The proliferation marker Ki67 was positive in a majority of tumour cell nuclei, in this area about 140 of 180 tumour cells (78%), which suggests a very high rate of proliferation. Any visible staining above background sufficed for positive classification of a tumour cell in stains with ER, PR and Ki67. ER oestrogen receptor. PR progesterone receptor. HER2 human epidermal growth factor receptor 2. Scale bars: A 200 µm. B 100 µm. ER, PR, HER2 and Ki67 40 µm.

As seen in Table 3, 73% of available primary tumours and 83% of metastases were positive for ER. Only 6 of 11 (55%) available primary tumours and 11 of 30 (37%) available metastases were positive for PR. As a result, 23 of 32 metastases (77%) were of the Luminal B subtype (Fig. 1).

**Table 3:** Surrogate subtypes of ocular and periocular breast cancer metastases.

	Primary tumours	Metastases
	*n* (% of available,% of all 32)	
**ER IHC**		
Positive^a^	8 (73, 25)	25 (83, 78)
Negative	3 (27, 9)	5 (17, 16)
**PR IHC**		
Positive^b^	6 (55, 19)	11 (37, 34)
Negative	5 (46, 16)	19 (63, 59)
**HER2 IHC**		
Negative	7 (64, 22)	11 (42, 34)
Equivocal		2 (8, 6)
Positive^c^	4 (36, 13)	13 (50, 41)
**HER2 FISH**		
Negative		5 (63, 16)
Positive		3 (38, 9)
**HER2 in either IHC or FISH**		
Negative	7 (64, 22)	12 (46, 38)
Positive	4 (36, 13)	14 (54, 44)
**Ki67**		
High^d^	2 (33, 6)	5 (50, 16)
Low	4 (67, 13)	5 (50, 16)
**Surrogate subtype**		
Luminal A	4 (40, 13)	2 (7, 6)
Luminal B	3 (30, 9)	23 (77, 72)
HER2 enriched	2 (20, 6)	1 (3, 3)
Triple negative	1 (10, 3)	4 (13, 13)

*ER* Oestrogen receptor, *PR* Progesterone receptor, *HER2* Human epidermal growth factor receptor 2, *IHC* Immunohistochemistry, *FISH* Fluorescence in situ hybridization.

^a^>1% of tumour cells positive for oestrogen receptors.

^b^≥20% of tumour cells positive for progesterone receptors.

^c^Circumferential HER2 membrane staining that is complete, intense, and in >10% of tumour cells.

^d^>25% of tumour cells positive for Ki67.

HER2 positivity was also highly represented, with 13 of 26 (50%) metastases being positive with HER2 IHC. Two metastases had equivocal HER2 staining. One of these two was also tested with FISH and turned out to be negative. Seven additional metastases were tested with HER2 FISH without prior HER2 IHC, of which 4 were negative and 3 positive. The total number of metastases with HER2 positivity in IHC or FISH was 14 (54% of 26, Table 3).

### Comparisons of biomarker expression and subtypes

**Fig. 2:**
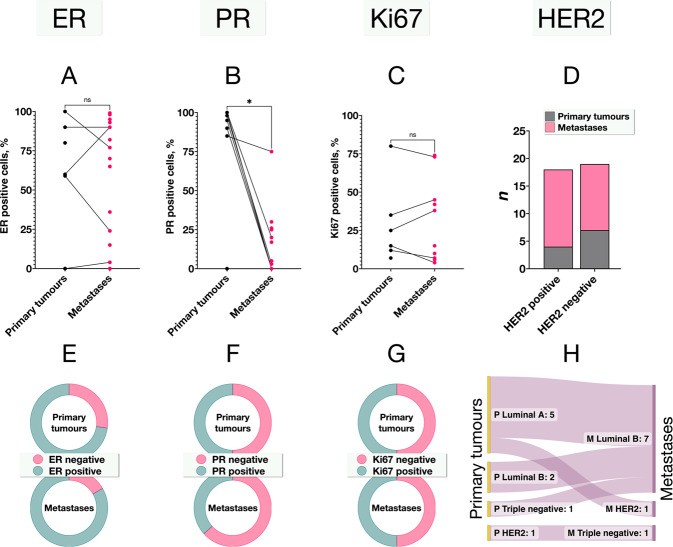
Biomarker expression in primary tumours and metastases. **A** There was no significant difference in the proportion of ER-positive tumour cells between primary tumours and metastases (Mann-Whitney *U P* = 0.61). **B** Metastases had a significantly lower proportion of PR-positive cells (*P* = 0.036). **C** There was no significant difference in the proportion of Ki67 positive cells (*P* = 0.94). In contingency tables, there were no significant differences in the distribution of **D** HER2 positive tumours (Fisher’s exact *P* = 0.48), **E** ER-positive tumours (≥1% of tumour cells ER positive, *P* = 0.66), **F** PR positive tumours (≥20% of tumour cells PR positive, *P* = 0.48), or **G** Ki67 positive tumours (>25% of tumour cells Ki67 positive, *P* = 0.63). **H** The distribution of the Luminal A, Luminal B, HER2 enriched and triple-negative subtypes did however differ between primary tumours and metastases, with the Luminal B subtype being overrepresented in metastases (*P* = 0.012). ER oestrogen receptor, PR progesterone receptor, HER2 human epidermal growth factor receptor 2. Lines in A to C indicate paired cases, with primary tumour and metastasis from the same patient. *P < 0.05. ns non-significant.

There was no significant difference in the proportion of ER-positive cells in primary tumours and metastases (Mann-Whitney *U P* = 0.61, Fig. 2A), but metastases had significantly lower proportions of PR positive cells (*P* = 0.036, Fig. 2B). The proliferation index as measured with the proportion of Ki67 positive tumour cells was similar in primary tumours and metastases (*P* = 0.94, Fig. 2C). In contingency tables, the distribution of HER2 positivity (Fisher’s exact *P* = 0.48, Fig. 2D), ER positivity (*P* = 0.66, Fig. 2E), PR positivity (*P* = 0.48, Fig. 2F) and Ki67 positivity (*P* = 0.63, Fig. 2G) was similar between primary tumour and metastases. The distribution of the Luminal A, Luminal B, HER2 enriched and triple-negative subtypes did however differ between primary tumours and metastases, with the Luminal B subtype being overrepresented in metastases (*P* = 0.012, Fig. 2H).

## Discussion

In this study, we demonstrate that more than half of breast cancer metastases in the eyes and orbits were positive for HER2, and that 23 of 32 metastases were of the Luminal B subtype. This could have important consequences for the management of patients with breast cancer metastases in this area, as there is a high chance that they can benefit from HER2-targeted therapy in addition to chemotherapy. We also show that biomarker status frequently changes between primary tumour and metastasis. Therefore, it is recommendable that metastases are biopsied before treatment decision are made. The high likelihood of HER2 positivity does not suggest that a treatment response to HER2-targeted therapy can be assumed without testing, and that biopsy rates should be reduced. Naturally, careful evaluation of a patient’s health and own wishes, extent of metastatic disease in other parts of the body, concurrent medications, and treatment response to previous regimens is required. Some choroidal metastases require a transvitreal approach, and although complications are rare, intraocular or deep orbital biopsies typically require general anaesthesia, significant resources and technical expertise [25–28].

Patients diagnosed with ocular breast cancer metastases typically receive systemic chemotherapy, hormonal therapy and external beam radiotherapy [17]. In most cases, the treatment is not expected to be curative [15–17]. In two recent publications, Blohmer et al. and Grajales-Alvarez et al. have outlined outcomes after treatment of orbital and periorbital breast cancer metastases in relation to histological growth patterns [29, 30]. A first distant metastasis with lobular histology entailed a significantly worse prognosis than ductal histology, but rates of HER2 positivity were lower and outcomes of HER2 targeted therapy were not reported.

None of the included metastases were sampled with fine needle aspiration cytology (FNAC). As shown previously, there may be marked variability between FNAC and other types of sampling methods, such as core needle biopsies [14, 31]. The latter allows for assessment of growth patterns in addition to the visual appearance of individual tumour cells, and has been shown to provide superior prognostic and treatment predictive information [23, 26]. We, therefore, recommend that ocular breast cancer metastases are sampled with methods that preserve tissue architecture, including incisional, excisional, and core needle biopsies.

This study has several limitations. Foremost, it is based on retrospective data from a limited number of metastases and even fewer primary tumours. This could reduce generalizability to the large number of breast cancer metastases that are examined in ophthalmic pathology laboratories worldwide. The risk of type I errors will also be increased. For example, the proportion of HER2 positive metastases of 54% observed herein is higher than in previous reports of metastases at other sites, but did not differ significantly from the 11 available primary tumors [4, 10, 11]. Secondly, the expression of the four examined biomarkers was assessed manually on slides stained at two different institutions over a period of 15 years, which might increase inter-rater and inter-method variability. Thirdly and last, the thresholds used for breast cancer surrogate subtype classification are not universal and are not used with global consensus. Other thresholds and definitions would have led to a different distribution of subtypes.

In conclusion, the Luminal B subtype and HER2 positivity are both highly represented in breast cancer metastases in the eyes and orbit. A large proportion of available cases switched subtype from primary tumour to metastasis. Future studies should evaluate these findings in a larger cohort.

## Summary

### What was known before

- Breast cancer is the most common cancer to metastasize to the choroid and orbit.
- Based on a set of biomarkers, breast cancer can be subdivided into four distinct subtypes with separate treatment and prognosis.
- The subtype is not necessarily identical in a primary tumour and its metastases.

### What this study adds

- For nearly one in four patients, the ocular metastasis is the first presentation of breast cancer.
- Metastases have significantly lower proportions of progesterone receptor positive cells than primary tumours.
- The distribution of breast cancer subtypes differ between primary tumours and metastases.
- Nearly 4 in 5 breast cancer metastases in the eyes and orbit are of the Luminal B subtype, and a small majority are HER2 positive.

## Data Availability

The datasets generated during and/or analysed during the current study are available from the corresponding author on reasonable request.
